# Mediastinal Eventration of a Pseudocyst of Pancreas Presenting As Acute Shock Syndrome: Expecting the Unexpected

**DOI:** 10.7759/cureus.21433

**Published:** 2022-01-19

**Authors:** Param Shah, Charan Bagga, Dhruv Talwar, Sunil Kumar, Sourya Acharya

**Affiliations:** 1 Department of Medicine, Jawaharlal Nehru Medical College, Datta Meghe Institute of Medical Sciences (Deemed to be University), Wardha, IND

**Keywords:** mediastinum, ct scan, pseudocyst, pancreatitis, diaphragm

## Abstract

Pancreatic pseudocyst is a usual complication of chronic pancreatitis. Diagnosis is usually established with the help of cross-sectional imaging. Typical presenting complaints are abdominal pain and vomiting. However, atypical presentations of pseudocyst of the pancreas continue to puzzle clinicians throughout the world, leading to difficulty in diagnosis and hence, the development of life-threatening complications. Here, we report a case of a 47-year-old male who was a known case of chronic pancreatitis related to alcoholism presenting with dyspnea, dysphagia, chest pain, and vomiting with a blood pressure of 70/50 mmHg, which upon evaluation revealed to be a case of peripancreatic pseudocysts extending into mediastinum abutting inferior vena cava and right atrium presenting as acute shock syndrome. The patient was managed with ultrasound-guided pigtail insertion and drainage of pseudocyst of pancreas. Eventually, the patient’s clinical condition did not allow for surgical exploration of the thorax and the patient succumbed.

## Introduction

Pseudocyst of the pancreas is a usual complication of chronic pancreatitis with the incidence rates ranging from 20% to 40% [1]. Chronic pancreatitis due to chronic alcoholism is found to be the most common causative factor. It is an encapsulated collection of fluid with a well-defined inflammatory wall, usually outside the pancreas with minimal or no necrosis. Rarely, pseudocyst can pass through the hiatus in the diaphragm and reach the mediastinal area; such patients present with a triad of dyspnea, dysphagia, and chest pain [2]. These complaints usually point towards various cardiac, pulmonary, and gastrointestinal disorders. A clinical presentation without the complaint of abdominal pain makes the diagnosis of pseudocyst of pancreas difficult and its extension into mediastinum presenting as a shock is not even a remote possibility. A definitive diagnosis is obtained through imaging modalities like computed tomography or magnetic resonance imaging showing cystic lesions extending from the peri-pancreatic area to the mediastinum [3]. Here we report a case of a 47-year-old male with chronic pancreatitis with multiple pseudocysts extending into the mediastinum, leading to shock.

## Case presentation

A 47-year-old male who was a known case of chronic pancreatitis presented to us with chief complaints of breathlessness for 15 days which progressed from NYHA Grade III to NYHA Grade IV with no exacerbating and relieving factors. The patient had dysphagia for the last ten days, which was mainly for liquids. The patient also had complaints of sudden onset chest pain for two days which was localized to the left side of the sternum, radiating to the back. The patient had non-bilious vomiting for two days. The patient had been abstinent from alcohol for eight months.

On general examination, his pulse was 108/min, BP was 70/50mmHg, oxygen saturation on room air was 94%, respiratory rate was 26/min, jugular venous pressure (JVP) was raised at 6 cm of water, with negative abdominojugular reflux with no edema feet and with no pallor and icterus. On abdominal examination, there was guarding and rigidity. On respiratory examination, there was reduced air entry in the left infrascapular area. CVS examination revealed no abnormality.

Blood parameters revealed low haemoglobin, increased INR, increased serum lipase, low serum calcium, increased C-Reactive Protein, and arterial blood gas analysis revealed metabolic acidosis with compensated respiratory alkalosis (Table 1).

**Table 1:** Showing laboratory investigations of the case

Laboratory investigations	Value	Biological reference range
Haemoglobin	10.2 g/dl	13-15 g/dl
Mean Copurscular Volume	102 fL	79-100 fL
Total Leukocyte Count	7300 /cumm	4000-11,000 /cumm
Platelet count	2,82,000 /cumm	1,50,000 – 4,50,000/ cumm
PT-INR	1.28	<=1.1
Serum Amylase	206 IU/L	30-110 IU/L
Serum Lipase	1289 IU/L	10-140 IU/L
Serum Calcium	6.8 g/dl	8.5 – 10 g/dl
Serum Urea	27 mg/dl	5-20 mg/dl
Serum Creatinine	0.4 mg/dl	0.3- 1.2 mg/dl
Serum Sodium	128 mmol/L	135-145 mmol/L
Serum Potassium	3.4 mmol/L	3.5-5.5 mmol/L
Serum Albumin	1.7 g/dl	3.5 - 5.5 g/dl
C-Reactive Protein	16 mg/L	<= 5 mg/L

By making a presumptive diagnosis of acute shock with acute on chronic pancreatitis, the patient was started on antibiotics, inotropic supports, aggressive fluid strategy, and injection octreotide. A 2D echo revealed normal biventricular function with normal collapsing IVC. Chest X-ray revealed mediastinal widening with obliteration of diaphragmatic outline.

**Figure 1:**
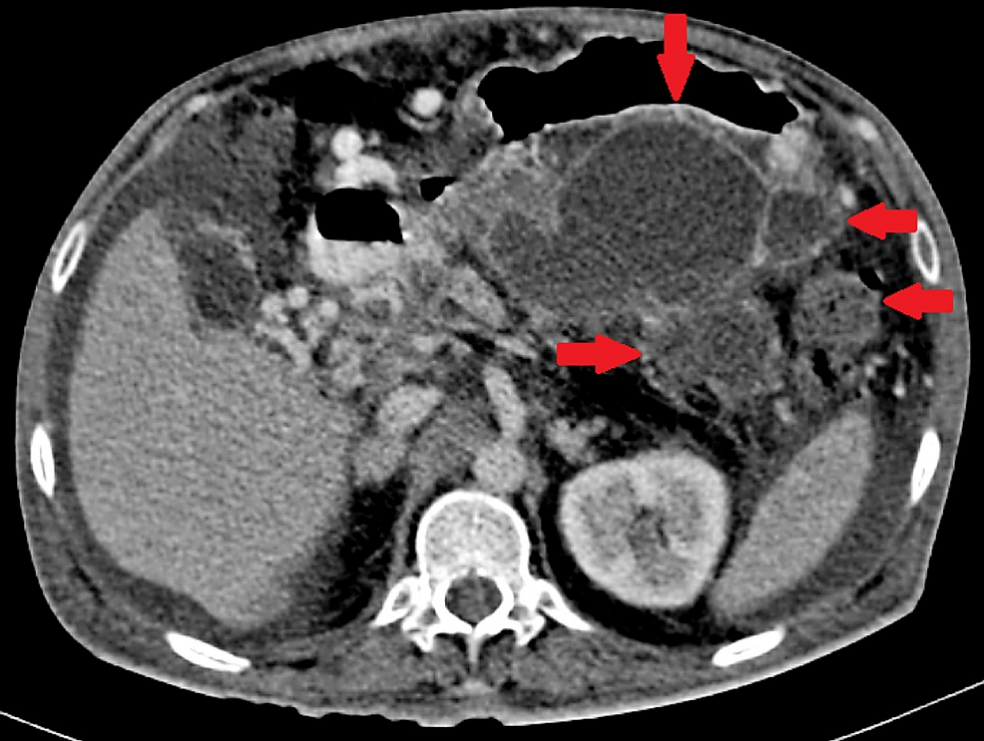
Contrast-enhanced computed tomography of the abdomen showing multiple pancreatic pseudocysts in the abdomen

**Figure 2:**
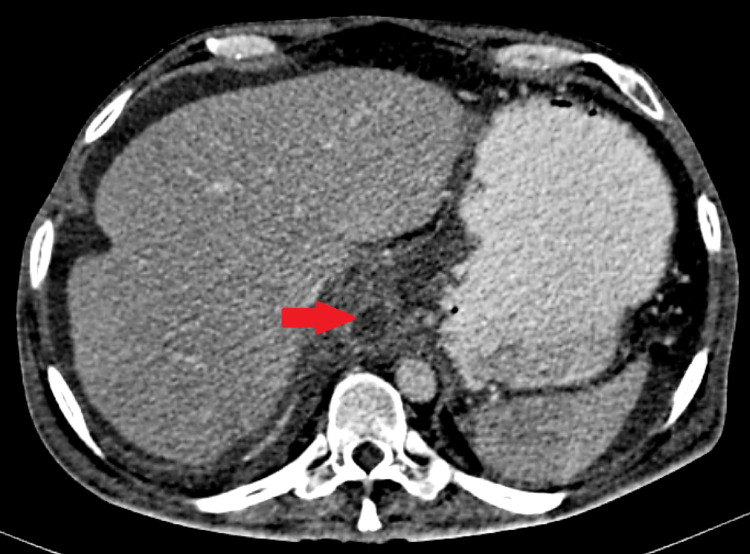
Contrast-enhanced computed tomography of the abdomen showing pseudocyst just below the diaphragm

On further evaluation, Contrast-Enhanced Computed Tomography of the abdomen was done, which revealed multiple thin-walled hypodense cystic lesions along the body and tail of pancreas, largest of size 8.8cm x 5.4cm with evidence of cystic lesions in aortocaval space abutting the inferior vena cava (Figure 1, 2).

**Figure 3:**
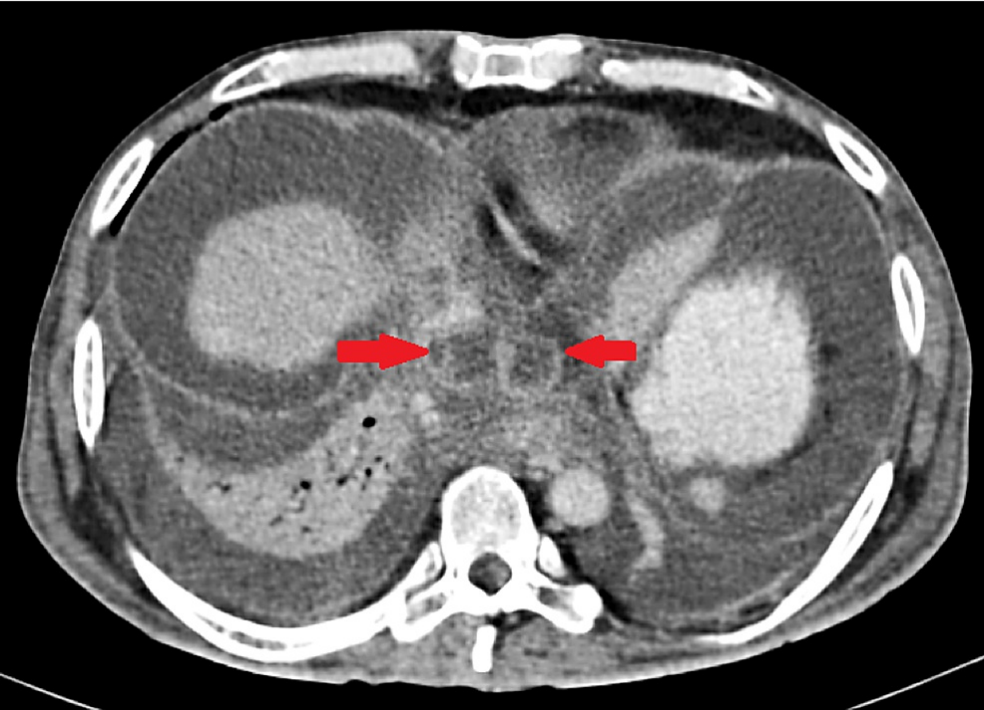
Contrast-enhanced computed tomography of the abdomen showing pseudocyst at diaphragm level, traversing the hiatus

**Figure 4:**
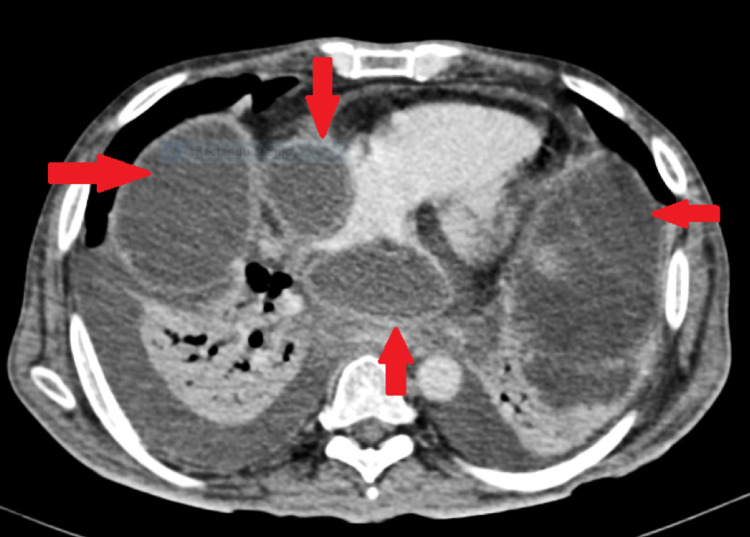
Contrast-enhanced computed tomography of the abdomen showing pseudocyst in the mediastinum, abutting right atrium.

HRCT of the thorax revealed a cystic lesion in the para-cardiac region extending and communicating with the anterior mediastinal region and middle mediastinum, abutting the right atrium and further extending into the upper abdomen through oesophageal hiatus (Figure 3, 4).

Pigtail catheterization was done into the largest pseudocyst of the abdomen through radiological guidance. The patient was further planned for an operative procedure for the management of multiple mediastinal pseudocysts compressing the right atrium. Due to rapid decompensation of clinical condition, further intervention could not be done and the patient died after two days of hospital stay. 

## Discussion

A formation of pseudocyst is a known complication of both acute and chronic pancreatitis. A pseudocyst is a collection of fluid around the pancreas, which is enclosed by reactive granulation or fibrous tissue. Most of the pseudocysts are localized to the peripancreatic area and they rarely reach the mediastinum. The pathogenic mechanism causing difficulty in breathing in a case of pancreatitis is usually due to the development of pleural effusion (usually left-sided). However, in very rare cases, pancreatic-pleural fistula and/or mediastinal extension of pseudocyst might be the underlying cause [4].

It is hypothesized that mediastinal pseudocysts are caused by rupture of the pancreatic duct posteriorly into the retroperitoneal space. The pancreatic fluid then lurches through the diaphragmatic hiatus into the mediastinum. Esophageal and aortic openings in the diaphragm are usual sites for entry of the pseudocyst into the mediastinum [5].

The fluid has different routes of traveling through the diaphragm into the mediastinum. Fluid primarily travels through the esophageal and aortic hiatus into the posterior mediastinum. However, if the diaphragm is penetrated through the inferior vena cava hiatus or the foramen of Morgagni, then this fluid shall extend into the middle and anterior mediastinum [6].

Atypical manifestations of the pseudocyst of the pancreas have been reported before [7-9]. However, the extension of pancreatic pseudocyst in the mediastinum is a rare outcome of alcohol-related pancreatitis with potentially life-threatening complications such as shock in our case and a challenging treatment course [10].

A multidimensional approach is required for attaining an optimal outcome. CT scan and MRI are required imaging modalities. Later on, the treatment modality has to be chosen after careful consideration of the extension and location of the pseudocyst of the pancreas [11].

Our patient presented with acute shock syndrome; however, the etiology of the same was not known. Mediastinal pseudocyst as an extension of pseudocyst of the pancreas, which was abutting right atrium and causing underfilling of right atria was ultimately singled out as the causative factor in the present case. Hence, higher suspicion of a lethal mediastinal extension of pseudocyst of the pancreas is needed in cases of pancreatitis who present with the triad of dyspnea, dysphagia, and chest pain [12].

## Conclusions

Mediastinal extension of a pseudocyst of the pancreas, though rare, is a life-threatening complication of chronic pancreatitis. Timely diagnosis of extension of pseudocyst of the pancreas into the mediastinum can alter the course of further management and prevent mortality. 
